# Polysaccharide-based hydrogel enriched by epidermal growth factor peptide fragment for improving the wound healing process

**DOI:** 10.1016/j.heliyon.2023.e22749

**Published:** 2023-11-23

**Authors:** Maryam Keykhaee, Farazaneh Sorouri, Mahban Rahimifard, Maryam Baeeri, Alireza Forumadi, Loghman Firoozpour, Mehdi Khoobi

**Affiliations:** a Department of Pharmaceutical Biomaterials and Medical Biomaterial Research Center (MBRC), Faculty of Pharmacy, Tehran University of Medical Sciences, Tehran, Iran; b Pharmaceutical Sciences Research Center (PSRC), The Institute of Pharmaceutical Sciences (TIPS), Tehran University of Medical Sciences, Tehran, Iran; c Department of Medicinal Chemistry, Faculty of Pharmacy, Tehran University of Medical Sciences, Tehran, Iran; d Department of Radiopharmacy, Faculty of Pharmacy, Tehran University of Medical Sciences, Tehran, Iran

**Keywords:** Polysaccharide-based hydrogel, Alginate, Gum Arabic, (S-acetamidomethyl Cys20, 31-EGF) peptide, Wound healing

## Abstract

Wounds represent a "silent epidemic" in the global population that impact significantly people's quality of life and the economy of societies. Owing to the numerous therapies, the pursuit of a perfect wound dressing with superior performance for treating all sorts of wounds is still underway. Several studies have shown the potential of integrating restorative peptides into the scaffolds as potential therapeutic candidates for wound healing. So far, there is little information about the wound-healing effect of S-acetamidomethyl Cys 20-31-EGF peptide, a main fragment of epidermal growth factor. In this regard, the effectiveness of this peptide in the alginate-gum arabic polysaccharide hydrogel was evaluated as a wound dressing (AG-P). Physicochemical evaluation of the hydrogels demonstrated that the incorporation of the peptide compressed the hydrogel network due to the presence of hydrogen and electrostatic bonds without significant effect on the mechanical, viscoelastic properties, swelling and degradation rate of the hydrogel. The hydrogel could continuously release the peptide and prevent rapid attenuation of its concentration. Cellular assessment of AG-P by scratch test and CFSE cytoplasmic dye/flow cytometry technique encouraged the migration and proliferation of human fibroblast cells, respectively. The effect of the AG-P on the expression of IL-6, TNF-α, NF-kB1 and VEGF genes indicated that this hydrogel reduced inflammation, and significantly increased angiogenesis compared to the control group based on the Real-time PCR results. *In vitro* assessment indicated that this structure can promote efficient and faster wound regeneration by altering the microenvironment of the wound. The hydrogel showed interesting features to be more equipped with other therapeutic agents making it as suitable dressing for various type of the wounds.

## 1 Introduction

wounds represent a "silent epidemic" in the global population with significant social and economic impact, affecting millions of people's quality of life [1]. Wound formation and loss of skin is a serious threat to the health of the human because thermal insulation, retention of body fluids and protection against external pathogens depend on the function of the skin [2]. An intricate and highly coordinated sequence of molecular and cellular processes allows the skin to demonstrate a high capacity for repair. When normal healing processes are disrupted, wounds may cease to heal, resulting in chronic wounds [3]. Generally, wound healing is separated into four consecutive and overlapping phases including homeostasis, inflammation, proliferation, and tissue remodeling. These processes may be hampered by persistent inflammation, impaired vascularization, changing the phenotypic and functional of the cells, as well as matrix degradation in abnormal wound repair. The ultimate goal of healing is to restore the integrity of the dermo-epidermal layers by enhancing proliferation, migration, and accurate functions of fibroblasts and keratinocytes [4,5]. Achieving this goal requires a complex synchronization of cytokines, chemokines, and different growth factors in successive stages in the wound microenvironment. Among these, growth factors have a special effect on regulating the growth and fate of the tissues by triggering cell signals and regulating cell function [6]. The growth factors includes platelet-derived growth factor (PDGF), fibroblast growth factor (FGF), transforming growth factor beta 1 (TGF-β1), keratinocyte growth factor (KGF), vascular epithelial growth factor (VEGF), epidermal growth factor (EGF), and etc., promoting the cell differentiation, migration, and proliferation within the treatment process [6].

EGF as a prevalent mitogenic factor through stimulating the migration and proliferation of the skin cells, leading to accelerating of granulation tissue and formation of epidermis [7,8]. Exogenous EGF treatment and application of EGF-containing gel around lesions enhanced re-epithelialization, decreased the risk of infection, and increased rate of wound regeneration [8,9]. Despite being a promising molecule for skin therapeutic application, poor EGF's permeability to the skin (∼Mw ≥ 6000 Da) limits its use in topical therapies [10,11]. Furthermore, its low half-life in the lesion area as a result of the high activity of proteases, its low yield of preparation from human bases, the high cost of its preparation and purification processes are other obstacles on using this potent growth factor [12,13]. On the other hand, traditional GF delivery need high doses and/or long periods of boosters which may lead to serious side effects [14]. Also, suitable delivery system would regulate the EGF release profile in a wound. Although intralesional injections and ointments has been already existed in the market; however, they are still inadequate products due to defective diffusion of EGF, low shelf-life, and bioavailability [15].

Therefore, a small molecule imitating EGF's effects by influencing the certain cell signaling pathways could be effective in wound repair [16]. Based on this, a variety of wound-healing peptides have been unveiled during recent years, showing significant potential for wounds management. One of the strategies in finding effective peptide sequence is to examine the structure of the proteins in body to obtain their effective therapeutic fragments [17]. Salivary histatin, an effective wound healing peptide, showed that its activity relies heavily on the region containing residues 20–32 (SHREFPFYGDYGS) [18]. Also, TP-508 (Chrysalin®), a Coagulation-related peptide section of the thrombin receptor binding domain, revealed 72 % complete recovery compared to the placebo group in diabetic ulcer with no side effects [19]. Additionally, human platelet-rich plasma and extracellular matrix-derived peptides (UN3 and Comb1) could improve diabetic wound healing through increasing angiogenesis and re-epithelialization [20]. As well as the aforementioned studies, a synthetic agonist of formyl peptide receptor 2 (WKYMVM) has been shown to accelerate re-epithelialization, new blood vessels and granulation tissue formation, suggesting that it might be useful in treating skin wounds [21]. The crystal structure of EGF (a 53-residue polypeptide) revealed six cysteine residues that form native intermolecular disulfide bonds and divide the protein into three domains of the A-loop (Cys 6 and Cys20), B-loop (Cys 14 and Cys 31), and C-loop (Cys 33 and Cys 42) [[22], [23], [24]]. Residues of B-loop (Met 21, Ile 23 and Leu 26) involving particularly in the interaction of extracellular region of EGF receptor (EGFR) contain domain I (One EGF binding site), hydrophobically. By EGF binding to EGFR with high affinity, receptor dimerization and signal transduction occurs, which consequently leads to DNA synthesis and cell proliferation [25,26]. Previous study showed amino acid sequences 20–31 of the EGF B loop fragment, (S-acetamidomethyl Cys20, 31-EGF) peptide, produced mitogenic and angiogenic effects like its parent molecule [27].

According to the studies, hydrogels have been potentially considered as appreciated biomaterials for topical delivery of peptides in wound areas. Local administration of a connexin43 peptide (ACT1 peptide, 25 amino acids) embedded in a hydrogel, accelerated re-epithelialization without toxic effects for diabetic wound treatment [28]. In another study, a chitosan-collagen hydrogel attached with QHREDGS, an angiopoietin-1 derivative peptide, improved angiogenesis and keratinocyte migration and accelerated chronic wound closure [29].

Generally, hydrogel-based dressings are used extensively in wound management because they have high exudate absorption rates, permit gas exchange (O_2_/CO_2_), and reduce wound bed temperature. The strong hydrophilic properties of hydrogels also allow them to maintain a moist environment, relieving the patient pain during removal [30]. In this context, polysaccharide-based hydrogels such as alginate, cellulose, chitosan, hyaluronic acid provide an unprecedented opportunity in wound regeneration due to their hydrophilicity, abundance, biocompatibility, and biodegradability [31]. This natural polymers freely or in combination with other polysaccharides can absorb and retain water without compromising their structural integrity [32]. The positive outcomes of earlier investigations demonstrated that the hydrogel created by combining alginate (Alg) with gum Arabic (GA) offers a favorable bioactive cellular milieu for chronic wound healing. This hydrogel has “suturing” effect at the wound site and provides moisture-rich microenvironment that motivate the survival, proliferation, and differentiation of cells and significantly promote wound re-epithelialization, collagen deposition, and would closure. The mucilage of the sundew plant (Drosera) served as a model for this hydrogel, and its gelation mechanism has been attributed to the cross-linking of polysaccharides through electrostatic contact between the acidic groups of the polysaccharides and calcium ions [33,34]. Calcium ions as a key modulator in skin cell function, has an established function in the regulation of skin homeostasis due to its modulation of cell lipid barrier function, proliferation, and maturation [33,35].

Alg, an anionic polysaccharide derived from marine source algae, has a high absorption capacity and can therefore be successfully used for the treatment of wounds with a high exudate level [36,37]. Moreover, it increases the production of collagen type I and angiogenesis, lowers the pro-inflammatory mediators level in the wound bed, and promotes tissue regeneration [38,39]. GA is also a weakly acidic polysaccharide, which is mainly composed of galactose (39–42 %) and arabinose (24–27 %) and trace amounts of other polysaccharides and protein [29]. The antibacterial and antioxidant effects of GA resulted in the improved wound healing process and showed promising effect for the treatment of intestinal mucosal inflammation [31]. The porosity of the Alg-GA hydrogel also offers a unique support for immobilization of therapeutic compounds like peptides, optimizing their sustained release at the wound site [33,40,41].

Considering the effectiveness of EGF and the obstacles to its use in wound treatment, little information about the effect of the peptide S-acetamidomethyl Cys 20-31-EGF as EGF's active loop and the importance of peptide-containing dressings, we focused on the preparation and biological evaluation of S-acetamidomethyl Cys 20-31-EGF immobilized Alg-GA hydrogel (AG-P) for wound regeneration. The chemical structure, morphology, porosity, swelling, degradation rate, and mechanical properties of the hydrogels were first investigated. We next assayed the biocompatibility, cellular proliferation, and their ability in the fibroblast cells migration. Finally, the effect of the AG-P on gene expression of VEGF, as the main angiogenesis marker and three important inflammatory markers (TNF-α, IL-6, and NF-κB1) was also evaluated.

## 2 Experimental section

### 2.1 Materials

Sodium alginate (SA), gum arabic (GA), S-acetamidomethyl Cys 20-31-EGF peptide, calcium chloride dihydrate (CaCl_2_.2H_2_O), DAPI (4′,6-Diamidino-2-phenylindole dihydrochloride) phosphate buffered saline (PBS), fetal bovine serum (FBS), streptomycin, carboxyfluorescein diacetate succinimidyl ester (CFSE), allantoein, dimethyl sulfoxide (DMSO) and Dulbecco's modified eagle's medium (DMEM), and mitomycin C, 3–4,5 Dimethylthiazol-2-yl-2,5-diphenyltetrazolium bromide (MTT) were acquired from Sigma-Aldrich Company (Munich, Germany). The human foreskin fibroblasts cell line (Hu02) was purchased from the cell bank of Pasteur Institute of Iran (NCBI). TM Total RNA Prep kit, 5X RT Pre-Mix, and 2X Real-Time PCR Master Mix (For SYBR Green I) were obtained from BioFact company (South Korea).

### 2.2 Preparation of the hydrogels (AG)

The AG hydrogel was fabricated based on the previous studies [33]. SA was cross-linked with GA through calcium ions. Separately, a solution of GA and SA in deionized water (500 μL) were prepared (3 % w/v, ∼0.03 mg/mL). The biopolymers were fully dissolved after 30 min of gentle stirring of the mixtures. Equal ratio of GA and SA solutions were combined and cross-linked with 1 mL of CaCl_2_ (20 mM) to prepare the hydrogel. Freeze-drying was employed to reach more sable product. For short-term storage, the hydrogel was maintained at 4 °C.

To fabricate AG-P hydrogel, 3 % w/v of SA was added to a solution of particular amount of the peptide (see section 3-1) in deionized water (500 μL). As stated above, 1 mL of CaCl_2_ (20 mM) was employed to prepare the target hydrogel, after addition of GA solution (3 % w/v) to the viscous P-SA solution.

### 2.3 Fourier transforms infrared (FTIR) spectroscopy

Structural characteristic of the AG hydrogel with and without P (EGF 20–31) was analyzed by FTIR spectrophotometer (Nicolet Magna 550) using the KBr disks.

### 2.4 Structural and morphological characterization of the hydrogels

The surface characteristics of the AG and AG-P was evaluated through field emission scanning electron microscopy (FE-SEM, VEGA-II TESCAN) by gold-coated samples. The mean pore size of the FE-SEM images was studied by Image J software. To characterize the elements in the hydrogels, energy dispersive x-ray spectrometer (EDS, S-4100, Hitachi, Japan) was used.

### 2.5 Porosity of the hydrogels

Hexane displacement method was applied to assess the porosity of the lyophilized hydrogels [42]. Accordingly, the hydrogels (5 mm × 5 mm × 1 mm) were immersed in a 10 mL graduated cylinder containing a certain volume of hexane. The following equation was used to determine the porosity of the prepared hydrogels. Here, V1 is the initial volume of hexane, V2 is the sum of the volume of hexane and immersed hydrogels, and V3 is the volume of hexane residual after removal of each hydrogel.

$$\text{Porosity}\left(\%\right)=\frac{V1-V3}{V2-V3}\times 100$$

### 2.6 Swelling and degradation ratio

The peptide-enriched and free AG hydrogel was weighed after freeze-drying and placed in 2 mL of PBS to characterize the water absorption capability [43]. To measure the swelling rate, the weight of the hydrogels was monitored at 24, 48, and 72 h.

The lyophilized samples were weighed and incubated in PBS at 37 °C to evaluate degradation rate. At predetermined every 5 days during the 25-day, the hydrogels were removed from PBS and dried. The weight of the samples was measured in each period of time after freeze-drying. Each analysis was carried out three times. The swelling and degradation rate of AG and AG-P were calculated as follow:

$$\text{Swelling}\;\text{ratio}\left(\%\right)=\frac{\text{Wt}-W0}{W0}\times 100$$

$$\text{Degradation}\;\text{ratio}\left(\%\right)=\frac{W0-\text{Wt}}{W0}\times 100$$

W_0_ is the weight of the initial dried gels (mg) and Wt is the weight of the gels swollen or degraded at different time points (mg).

### 2.7 Rheological and mechanical behaviors

The rheological property was studied by a rheometer (Physica MCR 302) with a frequency sweep analysis (strain = 10 % and angular frequency range = 1–100 rad/s) in PBS (pH 7.4 and room temperature). Storage and loss moduli (G′ and G″, respectively) were evaluated in the range of angular frequency. Moreover, the compression of the hydrogels was tested using a mechanical testing machine (STM-20, Santam). The hydrogels were prepared in the form of cylinders with a diameter of 13 mm and a length of 6 mm and then compressed at a speed of 1 mm min^−1^ at room temperature. The slope of the stress-strain curve in the linear region represents the compressive modulus.

### 2.8 The release of P (EGF 20–31) from AG-P hydrogel

To investigate the release of P (EGF 20–31), 300 μL of the prepared AG-P hydrogel containing 200 μg of the peptide was injected into the bottom of the vials and incubated in PBS (pH 7.4, 1 mL) at 37 °C. Sampling was performed at predetermined time points, and the medium was replaced with fresh medium. The released amount of P (EGF 20–31) in the medium was measured at 280 nm.

### 2.9 Cell viability assay of P (EGF 20–31) and the AG-P hydrogels

To obtain the optimal amount of the peptide, at first, the cytotoxicity of peptide solutions with different concentrations on human fibroblast cells (Hu02 cell line) was evaluated using the MTT assay [43]. Briefly, Hu02 cells seeded in a 96-well plate with a density of 10^4^ cells/well were cultured at 37 °C under 5 % CO_2_ for 24 h. The cells were then incubated with the various doses of the peptide (100, 200, and 400 μg/mL) for 24, 48, and 72 h. After incubation, the medium was withdrawn, and 20 μL of a 0.5 mg/mL MTT solution was added to the cells and they were next incubated for 4 h. In the next step, the formazan crystals were solubilized with 100 μL of DMSO for 30 min, and the absorption was measured using an ELISA reader (Synergy, Biotech Instruments Inc., Germany) at 570 nm. The cells treated with DMEM were considered as negative control, and AG and AG-P were the experimental groups. The test were carried out five times (n = 5). For the biocompatibility evaluation, the hydrogel extraction solutions were prepared [42]. The lyophilized gels were sterilized with 70 % ethanol for 30 min, washed three times with PBS, and exposed to UV radiation for 45 min. The sterilized hydrogels were immersed in 2 mL of DMEM and then located in an incubator at 37 °C for 3, 7, and 14 days. After preparing the extraction solutions, MTT assay was performed on Hu02 cells according to the above procedure.

### 2.10 Cell proliferation assay

Cell proliferation in the extraction solutions of free AG and AG-P were measure through CFSE cytoplasmic proliferation dye test and flow cytometry with FACS analysis [44]. The cultivated cells were washed with PBS, stained for 8 min at room temperature with CFSE (5 μM), and then CFSE was inactivated by FBS subsequently. CFSE-labeled cells were seeded in 6-well dishes after the remaining CFSE was removed by two series of washing with PBS. Next, the cells were incubated with the extraction solutions of AG and AG-P hydrogels, Mitomycin C (as negative control, 5 μg/mL), and allantoin (as positive control, 50 μg/mL) for 5 days. Using flow cytometry, the CFSE fluorescence intensity was evaluated. Each peak represents a new cell division generation.

### 2.11 Scratch-wound healing assay

Using the scratch test approach, the capacity of Hu02 cells to migrate into the injured region was evaluated. The cells were seeded in a 24-well plate (2 × 10^5^ cells/well) to make a confluent monolayer with approximate 75 % confluences [44]. Then, the plate's bottom was scratched to form a wound region using a 100 μL sterile pipette tip. After the cells washing by PBS to eliminate the debris, they were treated with the extracted solution of AG and AG-P hydrogels for 48 h. The cells were then stained with DAPI for 1 min after being fixed with 4 % paraformaldehyde for 15 min. Finally, the images were captured using a fluorescent microscope (Olympus BX51, Japan), and Image J software was used to quantify the change in the scratch area. All analyses were done in triplicate.

### 2.12 Real-time PCR analysis

The effect of the hydrogels on the expression of inflammatory genes TNF-α, IL-6, and NF-kB1 and VEGF as angiogenesis gene were analyzed by real-time PCR. BioFactTM Total RNA Prep kit were employed to extract total RNA from Hu02 cells in accordance with the manufacturer's instructions after 48 h of incubation in an extraction solution containing AG and AG-P hydrogels. The Thermo Scientific NanoDrop 2000c UV–Vis spectrophotometer (Thermo Scientific, USA) was used to measure the amount and concentration of RNA. An estimation of the purity of the nucleic acid was made using the ratio of OD260 nm/OD280 nm (the ratio in the range of 1.7–2.0). DNase-I and RNase-free kits was utilized to remove genomic DNA, and a BioFact™ cDNA synthesis kit was applied to reverse transcribe cDNA. The quantitative real-time PCR levels of each target gene were determined by SYBR green master mix and the Light Cycler 96 system (Roche). β-Actin as a common housekeeping gene and internal control was employed to normalize gene. The abbreviations and sequences of the primers lists in Table 1.

**Table 1:** Primers of β-Actin, IL-6, TNF-α, NFκ-B1, and VEGF for performing RT-PCR.

Gene name	Primer sequence (5′-3′)
β-Actin(forward)	TGGAACGGTGAAGGTGACAG
β-Actin(reverse)	AACAACGCATCTCATATTTGGAA
IL-6 (forward)	CGACAGCCACTCACCTCTTCAG
IL-6(reverse)	TTCTGCCAGTGCCTCTTTGCTG
TNF-alpha(forward)	GCCCATGTTGTAGCAAACCC
TNF-alpha(reverse)	TATCTCTCAGCTCCACGCCA
NF-κB1(forward)	AGCAAATAGACGAGCTCCGA
NF-κB1 (reverse)	ACGCAGTGGAATTTTAGGGC
VEGF (forward)	CTACCTCCACCATGCCAAGT
VEGF (reverse)	CGAGTAGCTGCGCTGATAGA

### 2.13 Statistical analysis

Statistical comparisons were carried out by using one and two-way ANOVA with GraphPad Prism 9. All quantitative results are presented as mean ± SEM. Statistical significance was defined as a P value of less than 0.05 (p < 0.05).

## 3 Results and discussions

### 3.1 Preparation and characterization of hydrogels

**Fig. 1:**
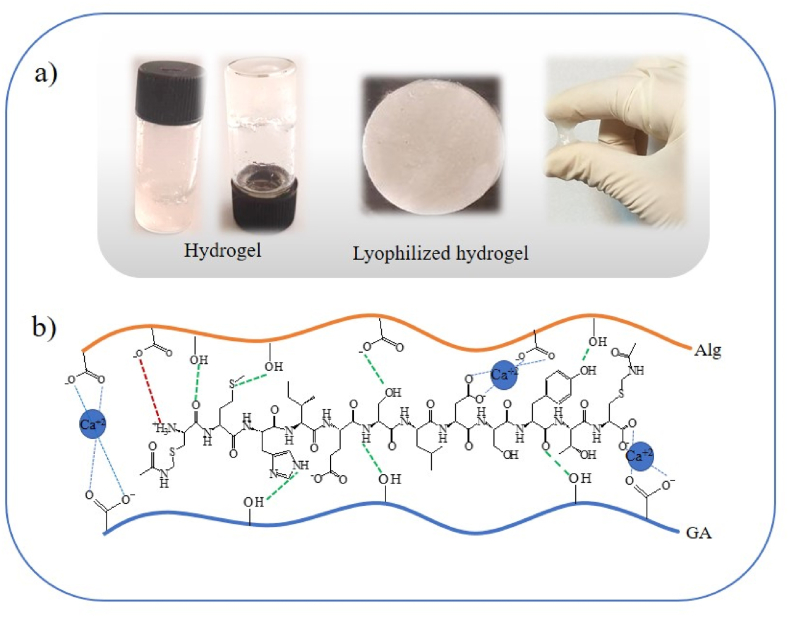
a) The morphology of freeze-dried AG-p hydrogel and its appearance after gelation. (b) Possible bonds (S-acetamidomethyl Cys20, 31-EGF) peptide and polysaccharide polymers in the AG-P hydrogel formation mechanism.

**Fig. 2:**
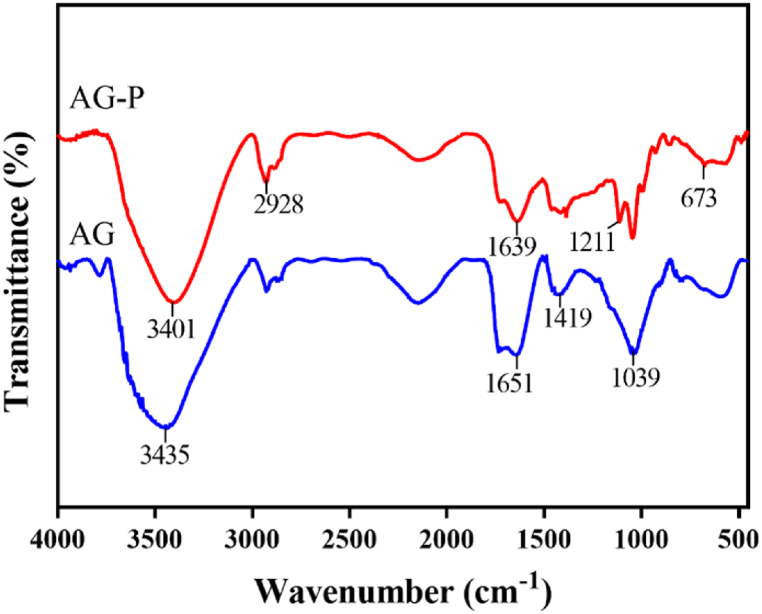
FTIR spectra of AG and AG-P hydrogels.

The AG hydrogel with and without P (EGF 20–31) were prepared via crosslinking of SA and GA and the conventional external gelation mechanism by adding CaCl_2_ as the source of Ca^+2^ (Fig. 1a and b) [35]. The possible interaction between P and AG in AG-P hydrogel was studied by FTIR spectroscopy (Fig. 2). For AG, the characteristic peaks at 3435 cm^−1^ and 1651 cm^−1^ were related to hydroxyl group and COO^−^ groups, respectively. The band recorded at 1039 cm^−1^ was due to the saccharide structure (C–*O*–C). The C–O bending of GA was assigned at 1419 cm-1, indicating the presence of both polymers in the AG hydrogel [45,46]. In the spectrum of AG-P, the signature peak at 3280–3401 cm^−1^, corresponding to the O–H and N–H stretching vibrations, was amplified significantly and revealed as a single broad peak. The red shifts of the O–H stretching vibration peak from 3435 to 3401 cm^−1^ and carboxylate anion from 1651 to 1639 cm^−1^ could be related to the hydrogen bonding and electrostatic interactions between P and AG polymer. The stretching vibrations of the C–H and N–H groups were observed at 2928 cm^−1^ and 673 cm^−1^, respectively. The band appeared at 1211 cm^−1^ could be corresponded to C–N stretching vibrations [47]. These results confirmed the successful enrichment of the AG hydrogel by P (EGF 20–31).

**Fig. 3:**
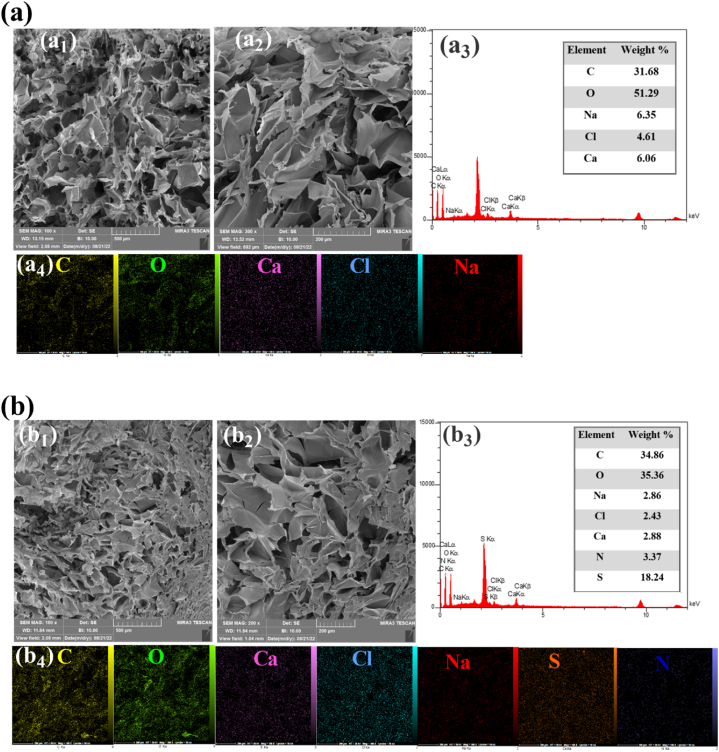
Characterization of AG and AG-P hydrogel. Different magnifications of FE-SEM images of AG (a1and a2) and AG-P (b1 and b2). EDS and elemental analyses of AG (a3 and a4) and AG-P (b3 and b4). Yellow, green, violet, blue, red, orange, and dark blue points represent C, O, Ca, Cl, Na, S, and N, respectively.

The morphology of the AG and AG-P (200 g/mL) hydrogels after fabrication was examined by FE-SEM (Fig. 3a1, a2, b1, and b2). Peptide trapping in the matrix of hydrogel made the AG-P structure more compact, which could be due to the successful integration of the peptide in the matrix of hydrogel, which leads to the creation of electrostatic interaction or hydrogen bonding between the peptide and hydrogel matrix. The hydrogels were further assessed by EDS analysis. EDS spectrum and the weight percentages of the components of free AG and AG-P showed the presence of C, O, Ca, Cl, Na, S, and N elements in different weight ratio (Fig. 3a3, b3). The nitrogen and sulfur peaks with weight percentage of 3.37 % and 18.24 % in the EDS spectrum of AG-P corroborated acceptable loading of the peptide. Also, the elemental mapping of the peptide-bearing hydrogel revealed a homogeneous distribution of the peptide (based on the distribution of S and N elements) in the hydrogel (Fig. 3a4, b4).

**Fig. 4:**
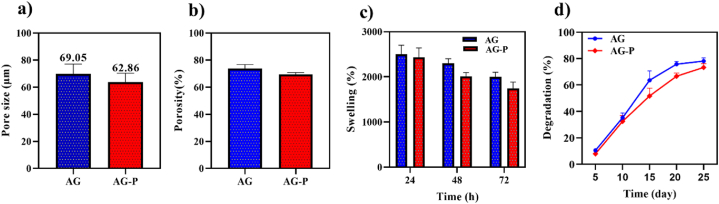
**a)** The mean pore size and **b)** porosity of the free AG and AG-P. **c)** The swelling ratio and **d)** degradation rate of AG hydrogel with and without P at 37 °C in PBS at various time interval.

The average pore size of AG and AG-P was measured using the micro-images of the FE-SEM and the results revealed the mean size of 69.05 ± 26.4 and 62.84 ± 33.7 μm, respectively (Fig. 4a). Furthermore, the average porosity of the hydrogels without and with P, based on the hexane displacement method, was ∼74 % and ∼69 %, respectively (Fig. 4b). Studies have shown that porous hydrogels promote cell proliferation and penetration, new blood vessel generation, and wound secretion absorption [48,49]. In wound dressings, the appropriate pore size for mammalian skin regeneration is 20–125 μm, and the optimal porosity for the cell infiltration is 60–90 % [43]. Since both AG and AG-P hydrogels exhibited acceptable pore sizes and porosities, they can be used for skin repair.

### 3.2 Swelling and degradation property of the hydrogels

A swollen hydrogel indicates liquid absorption caused by the hydrophilic nature of its constituents, which can be assessed by calculating the hydrogel's mass change [50]. In our study, the highest amount of swelling degree of AG-P hydrogel was rather similar to AG hydrogel in 24 h, indicated that the peptide did not change the water absorption capacity of the hydrogel (Fig. 4c). The presence of numerous carboxyl and hydroxyl groups in structure of the hydrogels offer suitable matrix for water interaction and retention. A swelling effect allows wound exudates to be absorbed [51]. The degradability of the scaffolds during the time affects drug release, and consequently the cell functions and tissue repair [52]. The degradation behavior of both lyophilized hydrogels was examined through measuring the mass loss in physiologically relevant buffer (PBS) condition up to 25 days. Peptide-free hydrogel lost more than 75 % of its initial weight during this time. The results revealed that addition of the peptide in the matrix of the hydrogel decreased the degradation potency of AG-P which could be probably due to the development of a denser porous construction in P-enriched hydrogel than P-free hydrogel (Fig. 4d). Although both samples lost weight, there were no drastic changes.

### 3.3 Rheological and mechanical properties

**Fig. 5:**
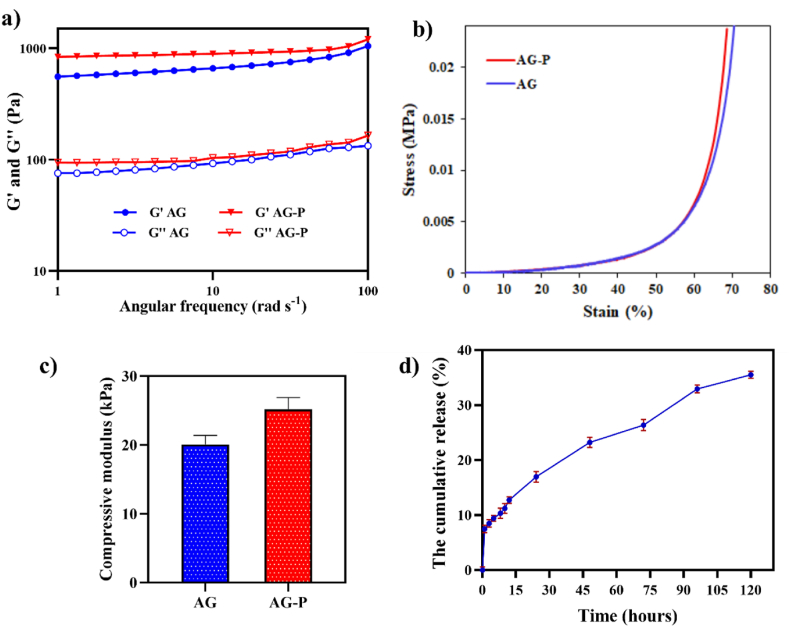
a) Angular frequency sweep test for AG and AG-P hydrogels. **b)** Stress–strain curves. **c)** The compressive modulus of the AG and AG-P hydrogels under compression; Data were analyzed by one-way ANOVA test, mean ± SEM, n = 3. d) The profile release of peptide from AG-P hydrogel.

Among the physical properties, the mechanical properties of hydrogels are the most important for their application in tissue engineering [53]. To determine the viscoelastic properties of AG hydrogel with and without P (EGF 20–31), an oscillatory rheology assessment was performed (Fig. 5a). G′ and G″ of AG and AG-P hydrogels were amplified by increasing in frequency, and the value of G′ was higher than G″ in the frequency range, indicating that the elasticity and stability of the hydrogel were retained after enrichment with peptide. Consistent with our findings, Hu et al. reported that the viscoelastic properties were not reduced by incorporating EGF in the matrix of hydrogels [54]. According to the compressive stress-strain evaluation (Fig. 5b), incorporation of the peptide in the AG hydrogel matrix showed similar stress in the same strain compared to the P-free hydrogel. The increase in the compressive modulus of the hydrogel in the presence of the peptide could be due to the interaction of the peptide with the functional groups of the polymer, although this improvement was not significant (Fig. 5c). However, Antonova et al. suggested that the incorporation of VEGF into polymer scaffolds improves their physical and mechanical properties [55]. The elasticity and mechanical behavior of AG-P hydrogel were also closer to skin tissue rather than that of before enrichment by peptide [56].

### 3.4 Release study

The release profile of the peptide embedded in the AG hydrogel is shown in Fig. 5d. The initial burst release during 24 h was due to the high swelling rate of the hydrogel in presence of the media. Then, release rate of the peptide from AG-P hydrogel decreased, which could be due to the low degradation rate of the AG-P hydrogel. After 5 days, only 35 % of the peptide was released. The hydrogel could continuously release the peptide and prevent its accumulation and rapid attenuation of the concentration at later times compared to when the peptide or growth factors are directly applied. Therefore, the peptide introduced into the hydrogel increases the time of its hydrolysis and thus prolongs the duration of the effect of the peptide [57].

### 3.5 Cytotoxicity assay of the peptide (EGF 20–31) and the AG-P hydrogels

**Fig. 6:**
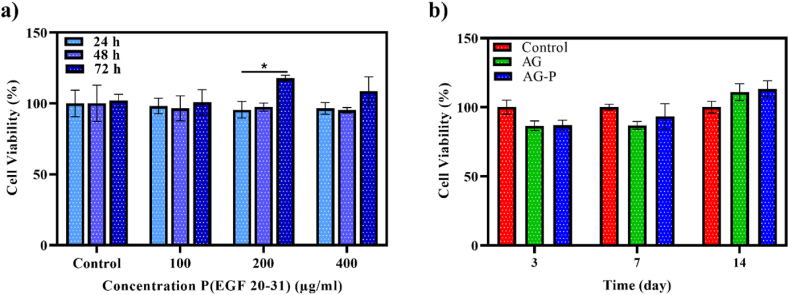
Cytotoxic assay of free P (EGF 20–31), free AG and AG-P hydrogels using MTT assay. a) Effect of various concentrations of P (EGF-2031) on the viability of Hu02 fibroblast cells (during 24, 48, and 72 h). b) Effect of extracted solutions of the hydrogels (on days 3rd, 7th, and 14th) on the viability of Hu02 fibroblast cells. * Shows significant differences from the control at P < 0.05. Bars represent the mean ± SEM, n = 6.

Cytotoxic effect of the peptide on Hu02 cells was assessed by MTT method. The cells were treated with different concentrations of the peptide for specific time period (24, 48, and 72 h) which showed no significant toxic effects in different doses and times (Fig. 6a). The highest percentage of viability (120 ± 0.65) was obtained at 200 μg/mL of the peptide confirming the efficient bioactivity of the prepared AG-P. Moreover, our study exhibited that the extracts of the hydrogels had no negative effect on the viability of fibroblast cells compared to the control (Fig. 6b). Also, the significant difference (P ≤ 0.05) in the AG-P (EGF 20–31) cell viability on day 14th compared to the days 3rd and 7th was observed which could be ascribed to the adequate release of the peptide from the hydrogel resulted from the appropriate decomposition rate of the hydrogels at this time.

Previous studies confirmed the biological activity of P (EGF 20–31) on fibroblast cells [27] and its N-terminal amino acid residues (YAC, 29–31) on keratinocyte cell proliferation [23]. Also, it was shown that AG does not have toxic effects on epidermal keratinocyte and dermal fibroblast [58].

### 3.6 Cell proliferation assay of the hydrogels

**Fig. 7:**
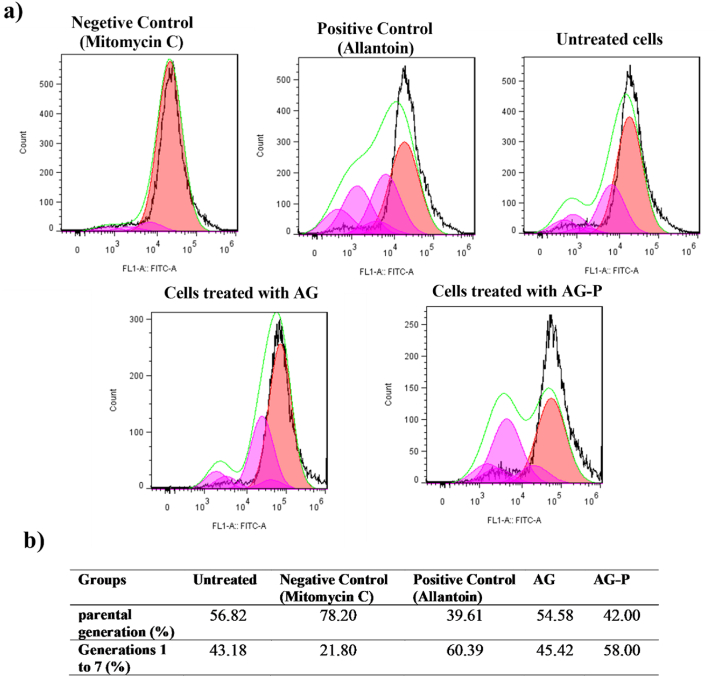
Impact of the AG and AG-P on the cell proliferation was assessed by flow cytometry. a) The samples' proliferative activity on day 5 is depicted in the histogram. b) Percentage of parental generation and percentage of generations 1 to 7 due to the untreated and treated cells with AG and AG-P extraction solution (200 μg/mL), Allantoin (50 μg/mL) and Mitomycin C (5 μg/mL) on day 5th.

The cell proliferation ability of the peptide trapped in the AG hydrogel was evaluated through flow cytometry and measuring the CFSE fluorescence intensity (Fig. 7a), which is an efficient technique for cell division screening [59]. We found that the proportion of proliferating cells significantly increased following treatment with AG-P, rising from 43.18 % in untreated cells to 58 % in treated cells, which was consistent with the outcomes when the cells were treated with allantoin as a positive control (50 g/mL) (Fig. 7b).

In prior research, it was observed that P (EGF 20–31) could stimulate DNA synthesis in the primary culture of human fibroblasts and displace 60 % of EGF receptors on the same cells [27]. Moreover, its mitogenic characteristics were confirmed on breast cancer cells [60].

### 3.7 Scratch-wound healing assay

**Fig. 8:**
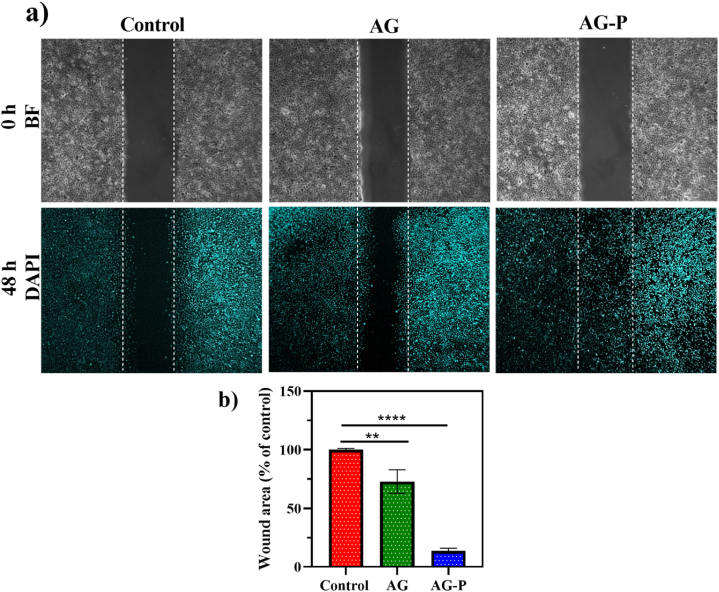
Scratch-wound healing assay. a) The fluorescence microscopy images of Hu02 migration after 48 h (BF and DAPI mean bright field and DAPI staining, magnification × 20). b) The quantification result of the wound area (% of Control) analyzed with Image J software. **, **** Significant differences from control at P < 0.01 and p < 0.0001, respectively. Bars represent the mean ± SEM of three experiments.

The *in vitro* scratch wound model is a repeatable method for examining cell migration and wound closure [42]. The migration effect of Hu02 cells after incubation with treatment groups for 48 h was evaluated (Fig. 8a). The results indicated that extraction of AG and AG-P structure improved the Hu02 migration from scratch's edge in comparison to the control group. The quantification result of the scratch area revealed that this effect was more noticeable in group AG-P than AG, nearly 95 % of the scratch was repaired (Fig. 8b). AG hydrogel enhanced fibroblasts and keratinocyte migration and promoted wound closure due to presence of Ca^2+^ in the hydrogel structure [61,62]. In another study, the ability of EGF to promote fibroblast cell migration has been observed [63]. It has been also reported that EGF could promote fibroblast cell migration through the recovered levels of EGF-receptor (EGFR) [64]. Since this peptide sequence is an active EGF fragment, it could play appropriately the role in fibroblast cell migration.

### 3.8 The effects of the hydrogels on the angiogenesis and inflammation gene expression

Impaired angiogenesis is a serious issue in failure of wound repair which deprives cells and tissues from nutrients and oxygen [65]. This issue causes vascular necrosis, incites inflammation, and inhibits epithelialization and extracellular matrix (ECM) creation. Therefore, improving angiogenesis in the injured area can be an ideal therapeutic strategy which is associated with increasing the expression of angiogenic factors as a result of rapid wound regeneration [3]. Angiogenesis encouraged granulation, and tissue remodeling during the hemostatic plug formation via TGF-β, fibroblast growth factor (FGF), and platelet-derived growth factor (PDGF). Vascular endothelial growth factor (VEGF) induces neovascularization against hypoxia and cytokines, and repairs damaged blood vessels. Fibroblasts from nearby undamaged skin tissue are accumulated in the wound area or they can be produced through differentiation of blood-borne circulating adult stem cells or precursors [66,67].

**Fig. 9:**
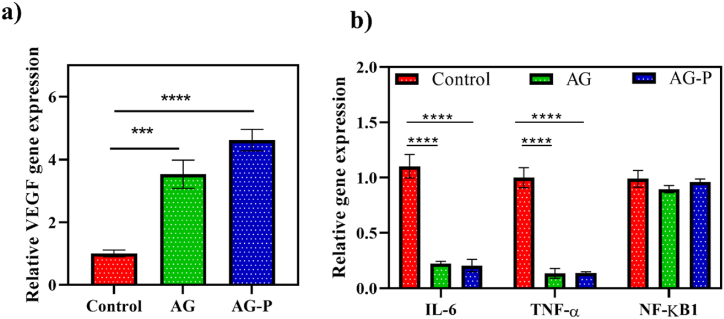
Gene expression analysis by real-time PCR. (a) Effect of the peptide addition (200 μg/mL) on VEGF gene expression (b) Impact of the peptide addition (200 μg/mL) on the expression of the inflammatory genes, IL-6, TNF-α, and NF-κB1. The inflammation and VEGF gene expression levels were normalized against β-Actin gene expression. *** And **** are significant differences from the control at P < 0.001, and P < 0.0001, respectively. Bars show the average ± SEM of three experiments.

Our finings exhibited that AG and AG-P efficiently improved VEGF expression as a potent angiogenic factor in the human skin fibroblast (Fig. 9a). However, the effect of AG-P (4.61 ± 0.26) was more noticeable than AG (3.53 ± 0.27). Calcium ion as an angiogenic stimulating factor promotes wound repair via the migration of dermis, epidermis cells and reorganization of endothelial progenitor cells [68]. Polymeric dressings containing calcium ions accelerated the wound healing process by suppressing inflammation, increasing angiogenesis and collagen synthesis [68,69]. EGF reduced wound healing times and increased re-epithelialization by improve angiogenesis through VEGF-mediated pathways [70]. In this study, it was also shown that P (EGF 20–31) enhanced angiogenesis, and its biological responses could be probably related to the stimulating EGF-related receptors [60].

In another approach, inflammation indicates the immune system's response to the external factors, microorganisms and damaged host tissue, and its management is necessary for effective treatment [71]. Irregular and longstanding inflammatory responses are the main provider of tissue dysfunction and ineffective wounds repair. Fibroblasts, keratinocytes, and immune cells along with several cellular and molecular mediators such as nuclear factor kappa B (NF-kB) pathway which is pivotal for controlling inflammation [44]. Tumor necrosis factor-alpha (TNF-α) and interleukin-6 (IL-6) are two main pro-inflammatory cytokines that cells may overexpress, disrupting the stages of wound healing and resulting in tissue damage and persistent inflammation [72]. By using the Real-Time PCR method, we assessed the expression of the IL-6, TNF-α, and NF-kB1 genes in this work. According to the findings, the AG and AG-P significantly decreased IL-6 and TNF-α levels compared to the control group (P < 0.0001) and showed a negligible reduction in NF-κB1 pathway (Fig. 9b). The behavior of the two hydrogels was similar in reducing the inflammatory factors, which indicated that AG structure played a key role in the anti-inflammatory effect. Alg suppressed the production of IL-1β, TNF-α, and other proinflammatory cytokines and blocked the NF-κB activation pathway [73]. Moreover, GA effected on the immune markers and can significantly diminish the level of TNF-α [74,75].

Comparing the results of this study with other previous studies [[[16], [17], [18], [19], [24], [25], [76]]indicated that this structure could probably contribute significantly in wound healing with the synergistic effect of the hydrogel and peptide through improving different phases of healing (Table 2).

**Table 2:** Peptide sequences derived from the active part of proteins in the body as immobilized or non-immobilized form in wound regeneration.

Name/Sequence peptide	peptide source	Concentration of peptide	Result	Ref
S-acetamidomethyl Cys 20-31-EGF peptide with alginate–gum arabic hydrogel	Epidermal growth factor	200 μg/ml	*In vitro* study: the structure improved the migration and proliferation fibroblast cells. The evaluation of the genes involved in the wound regeneration demonstrated that hydrogel reduced inflammation, and the presence of the peptide in the hydrogel increased angiogenesis significantly.	Current study
QHREDGS immobilized in chitosan–collagen	Angiopoietin-1	100 and 650 μM	*In vitro* and *in vivo* study: the structure enhanced the rate of re-epithelialization and the granulation tissue formation without affecting angiogenesis significantly.	25
SIKVAV -modified chitosan hydrogel	Laminin A protein	Not reported	*In vivo* study: the structure accelerated skin wound repair and re-epithelialization, as well as angiogenesis and collagen deposition. It promoted the secretion of EGF in skin ulcers.	76
SHREFPFYGDYGS	Salivary histatin	10 μM	*In vivo* study: The peptide did not encourage proliferation but induced cell spreading and migration.	16
Chrysalins (TP508)	Human thrombin	1–10 μg	The healing of diabetic foot ulcers was stimulated in a placebo-controlled phase I/II study.	17
Comb1+ (UN3)	Matrix- and plasma-derived peptides	1.0 mg/mL of Comb1 and 284 μg/mL of UN3	*In vivo* study: These peptides amplified steady-state mRNA levels, driving epithelialization, angiogenesis, and immune and progenitor cell chemotaxis.	18
WKYMVm	Formyl peptide receptor 2	1–100 μM	In vivo study: the peptide enhanced infiltration of immune cells and angiogenesis.	19
ACT1	Protein connexin43	100 μM	Clinical trial: the peptide accelerated wound re-epithelialization	24

## 4 Conclusion

Over the past years, various peptides have been isolated from existing protein structures that can play an effective role in wound repair. They offer a unique class of bioactive agents that can be prepared at a low cost with few immunogenicity issues. Peptides, as biological signals mimicking the growth factors, have a very important function in modulating cell activities such as survival, proliferation, migration, differentiation, and tissue regeneration. In this research, due to the pivotal role of epidermal growth factor in the wound regeneration process, the peptide (S-acetamidomethyl Cys20, 31-EGF) in the B loop of EGF, was immobilized into the matrix of the AG hydrogel and served as an appropriate dressing for wound repair. Hydrogel characterization showed that presence of the peptide had no significant effect on the physio-chemical properties of the hydrogel. The hydrogel showed appropriate ability to control the release of the peptide and prevent its rapid accumulation compared to the free peptide or growth factors. An *in vitro* study indicated that this structure increased the migration and proliferation of fibroblast cells without any toxicity. The evaluation of the genes involved in the wound regeneration demonstrated that AG and AG-P hydrogel reduced inflammation, and presence of the peptide in the hydrogel structure increased angiogenesis significantly due to strengthening EGFR signaling pathway and could control the rate of DNA synthesis. Our research offers a new approach for the preparation of peptide-based hydrogels that can be used topically for diverse skin wounds. We hope that these findings will open a new window in the treatment of EGF-related diseases such as acne, aging ulcers, glomerular kidney or chronic obstructive pulmonary diseases. Additionally, it seems that AG-P, in combination with other pharmaceutical agents or separately, can be used in angiogenesis and inflammation-related diseases, including diabetic retinopathy, autoimmune diseases, rheumatoid arthritis and atherosclerosis.

## Declarations

Conflict of interest the authors declare that there is no conflict of interest.

## Data Availability statement

Data will be made available on request.

## CRediT authorship contribution statement

**Maryam Keykhaee:** Writing – original draft, Software, Methodology, Formal analysis. **Farazaneh Sorouri:** Writing – review & editing, Methodology, Formal analysis, Data curation. **Mahban Rahimifard:** Writing – review & editing, Validation, Software, Methodology, Formal analysis. **Maryam Baeeri:** Writing – review & editing, Visualization, Validation, Software, Methodology, Formal analysis. **Alireza Forumadi:** Writing – review & editing, Resources, Data curation. **Loghman Firoozpour:** Writing – review & editing, Resources, Funding acquisition. **Mehdi Khoobi:** Writing – review & editing, Validation, Supervision, Investigation, Funding acquisition, Conceptualization.

## Declaration of competing interest

The authors declare that they have no known competing financial interests or personal relationships that could have appeared to influence the work reported in this paper.
